# Comparative Transcriptomics of Feather Follicles Reveals Potential Candidate Genes for Duck Feather Type Differentiation

**DOI:** 10.3390/ani16142267

**Published:** 2026-07-22

**Authors:** Wengui Wang, Jiangpeng Guo, Liang Wang, Meng Zhang, Xinye Zhang, Xiaoyu Jiang, Tairan Chen, Xiaohan Mei, Xufang Ren, Lujiang Qu

**Affiliations:** 1 National Engineering Laboratory for Animal Breeding, College of Animal Science and Technology, China Agricultural University, Beijing 100193, China; strawberrywhisky@cau.edu.cn (W.W.); xinye_leaf@163.com (X.Z.); jxy1581908148@163.com (X.J.); rachelchentairan@gmail.com (T.C.); 2022333020320@cau.edu.cn (X.M.); 2 Beijing General Station of Animal Husbandry, Beijing 100107, China; 3 Beijing Golden Star Duck Co., Ltd., Beijing 100076, China

**Keywords:** down feathers, contour feathers, feather follicles, transcriptome, differentially expressed genes, Beijing duck

## Abstract

Down feathers and contour feathers are two distinct feather types with vastly different structures and functions coexisting in the same individual. However, the molecular mechanism underlying these differences remains unclear. In this study, skin tissue containing intact feather follicles was collected from four anatomically defined regions of healthy Beijing ducks (*Anas platyrhynchos domesticus*) for transcriptomic sequencing: the breast and abdomen, which generate down feathers, and the wingtips and rump, which produce contour feathers. To mitigate bias arising from site-specific effects, we established paired comparisons between contour feathers and down feathers across different regions. Across all comparisons, 36 core differentially expressed genes (DEGs) were consistently identified. These encompassed nine *HOX* family transcription factors across three paralogous clusters, *ZIC* family members (*ZIC1* and *ZIC4*), *WNT4*, *TBX4*, *BMP5*, cytoskeletal and sarcomeric genes (*MYOZ2*, *ACTN2*, *DES*, *SMPX*), extracellular matrix regulators (*ASPN*, *MGP*, *FMOD*), and lipid metabolism-related genes (*MOGAT2*, *PNPLA2*), among others. Pathway enrichment analysis revealed that transcriptomic variations in different feather follicles involve gene modules functioning in positional identity, cytoskeletal organization, extracellular matrix remodeling and lipid metabolism, with potential neuroendocrine modulation. The transcriptomic dataset established here facilitates future studies on the molecular mechanisms of feather differentiation and offers a reference for molecular breeding to enhance down feather yield and quality of Beijing ducks.

## Simple Summary

Down feathers and contour feathers in ducks exhibit distinct morphological and structural characteristics. Down feathers are soft and fluffy and primarily function in thermal insulation, whereas contour feathers are relatively stiff and smooth and contribute to body protection, body shape maintenance, and aerodynamic performance. In this study, skin samples were collected from four body regions of healthy adult ducks: the breast and abdomen, which are enriched in down feathers, and the wing and rump regions, which are predominantly covered with contour feathers. Comparative transcriptomic analysis was conducted to investigate gene expression differences among feather follicles from these regions. A total of 36 candidate genes potentially associated with regional differences in feather follicle development and feather morphology were identified, including *WNT4*, *ZIC1*, *ZIC4*, *HOXC13*, *TBX4*, *BMP5*, *HOXC6*, and *HOXC10*. These findings provide insights into the molecular mechanisms underlying the formation of different feather types in birds and may contribute to future strategies for improving feather quality in poultry.

## 1. Introduction

Feathers are among the most structurally complex and functionally diverse keratinized skin appendages in vertebrates [1,2,3]. Within a single bird, two fundamentally distinct feather types coexist. Contour feathers (pennaceous), characterized by a rigid rachis and interlocking barbs that form a continuous vane for aerodynamic performance and protection, and down feathers (plumulaceous), which lack hooklet-mediated cohesion and instead form a loose, radially branched structure optimized for thermal insulation [4,5]. Despite their pronounced morphological and functional divergence, both feather types arise from homologous follicles sharing a common developmental origin and conserved structural framework [6,7,8].

Feather morphogenesis is governed by coordinated epithelial–mesenchymal interactions involving several conserved signaling pathways [9]. In avian skin, Wnt/β-catenin signaling regulates feather-specific morphogenetic processes, including feather tract and primordium formation, feather bud polarity, proximal–distal elongation, and follicle growth, as supported by studies on *WNT7A*, β-catenin activation, and Dickkopf-1-mediated pathway inhibition [10,11,12], while mammalian hair follicle studies further support its conserved role in skin appendage initiation and cycling [13,14,15]. BMP and TGF-β signaling pathways contribute to branching morphogenesis and periodic patterning [16,17,18], and Sonic Hedgehog (SHH) signaling further refines branching architecture [6,19,20]. In parallel, HOX genes encode positional information and contribute to regional specification of skin appendages [21,22,23]. Yet a fundamental question remains unresolved, whether the transcriptional differences observed between feather types reflect intrinsic, feather-type-specific regulatory programs, or are largely epiphenomenal to the positional identity of the skin region in which each follicle resides. This distinction is non-trivial, as skin transcriptomes inherently capture anatomical location in addition to follicle identity [24], making it difficult to determine whether pathways implicated in feather morphogenesis are organized into feather-type-specific regulatory networks or whether their apparent differential activity is primarily a consequence of regionally patterned transcriptional variation [25]. A systems-level understanding of the transcriptional programs that distinguish contour and down feather follicles across different anatomical regions is therefore still lacking [24,26,27].

The domestic duck provides a powerful system to disentangle these effects. Distinct feather types are spatially segregated within the same individual, with down-rich plumulaceous feathers localized to the breast and abdomen, and contour feathers occupying regions such as the wing tip and rump [24,28]. This organization enables within-individual comparisons that reduce inter-individual variability while preserving biologically relevant spatial context. Combined with a well-annotated reference genome and practical accessibility of tissue sampling, this makes duck an advantageous model for studying feather-type differentiation [29,30,31,32].

To address the confounding effects of anatomical origin, we implemented a multi-regional cross-comparative transcriptomic framework. Skin samples containing intact follicles were collected from four anatomical regions representing the two feather types, and multiple pairwise comparisons were constructed across regions. By identifying genes consistently differentially expressed across all comparisons, we defined a set of robust feather-type-associated transcriptional signatures that are repeatedly observed across anatomical contexts. Using this approach, we identified a conserved set of differentially expressed genes associated with feather type, including *WNT4*, *ZIC1*, *ZIC4*, *TBX4*, *BMP5* and multiple *HOX* family members, as well as a cytoskeleton-related gene module enriched in plumulaceous follicles. Pathway analysis further revealed that transcriptional differences among contour feather follicles are strongly influenced by anatomical origin, indicating previously unrecognized molecular heterogeneity within contour feather populations. These findings provide a multi-site transcriptomic framework for understanding feather type differentiation in ducks, highlight gene expression signatures associated with feather type identity for future functional investigation, and offer a foundation for the development of molecular breeding strategies aimed at enhancing down feather yield and quality in duck production.

## 2. Materials and Methods

### 2.1. Ethics Statement

This study was approved by the Animal Welfare Committee of China Agricultural University (approval number: AW42505202-1-05), and all ducks used in this study were handled according to the relevant regulations.

### 2.2. Collection of Animal Samples

This study selected six healthy Beijing ducks, including three males and three females, all 6 weeks old with similar body weights. The animals were purchased from Beijing Golden Star Duck Co., Ltd. (Beijing, China) and housed under standard conditions. Euthanasia was performed by cervical dislocation in accordance with approved protocols. Skin samples containing intact feather follicle structures were collected from four anatomical regions with different feather types, the breast and abdomen (down feather regions) and the wing tips and rump (contour feather regions). For each region, standardized skin tissue samples of approximately 1 cm^2^ containing intact feather follicles were collected using surgical scissors. Because the Beijing ducks used in this study were 6 weeks of age and undergoing the peak molting period, the feather shafts at the base were visibly engorged with pulp, indicating that the follicles were already in an active growth phase. Accordingly, the conventional step of plucking the feathers and waiting approximately 10 days for regrowth was omitted, and the emerging feathers were trimmed directly. Specifically, a 4 cm × 4 cm area of skin within each predefined sampling region was first disinfected with alcohol-soaked cotton, after which the feathers within this area were trimmed close to the skin surface using surgical scissors. Surface debris was then wiped away with alcohol-soaked cotton. Once the skin was lifted with forceps, a 1 cm × 1 cm section of skin was rapidly excised and rinsed in PBS prepared with DEPC-treated water, then transferred into a 1.5 mL centrifuge tube and stored in liquid nitrogen until further use. These samples were collected solely for the purpose of RNA extraction and transcriptomic sequencing. After collection, all samples were immediately frozen in liquid nitrogen and stored at −80 °C until further processing.

### 2.3. Total RNA Extraction and Quality Assessment

Approximately 50 mg of frozen skin tissue containing intact feather follicles was ground into a fine powder in liquid nitrogen. Total RNA was extracted using TRIzol reagent (Thermo Fisher Scientific, Waltham, MA, USA) according to the manufacturer’s instructions. Briefly, the powdered tissue was lysed in 1 mL of TRIzol reagent, followed by phase separation using chloroform. The aqueous phase containing RNA was collected, and the RNA precipitate was washed with 75% ethanol and dissolved in RNase-free water. RNA concentration and purity were determined using a NanoDrop spectrophotometer (Thermo Fisher Scientific), and RNA integrity was assessed using an Agilent 2100 Bioanalyzer (Agilent Technologies, Santa Clara, CA, USA). Only RNA samples with an RNA integrity number (RIN) of ≥7.0 and satisfactory purity were used for subsequent library construction.

### 2.4. mRNA Enrichment, Library Construction, and Sequencing

For each sample, polyadenylated messenger RNA was isolated from total RNA using mRNA Capture Beads (Vazyme, Nanjing, China). Two successive rounds of magnetic-bead purification were performed to increase the enrichment efficiency of poly(A)-containing mRNA. During the first round, total RNA was incubated with the mRNA capture beads to allow poly(A)+ RNA to bind to the magnetic beads. After washing, the captured mRNA was eluted at 80 °C using Tris buffer (Vazyme, Nanjing, China). The eluted mRNA was then rebound to the magnetic beads, washed, and eluted again using Tris-HCl buffer (Vazyme, Nanjing, China). The purified mRNA was fragmented by incubation with First Strand Synthesis Reaction Buffer. Random primers were added during fragmentation and used for first-strand cDNA synthesis. First-strand cDNA was synthesized by reverse transcription, followed by second-strand cDNA synthesis. The resulting double-stranded cDNA was purified using 1.8× DNA Clean Beads (Vazyme, Nanjing, China). RNA-seq libraries were prepared using the NEBNext^®^ Ultra™ RNA Library Prep Kit (New England Biolabs, Ipswich, MA, USA). The purified double-stranded cDNA was subjected to end repair and 3′-end adenylation, followed immediately by sequencing-adapter ligation. USER enzyme treatment was subsequently performed to open the adapter structure. Adapter-ligated products were size-selected using magnetic beads, amplified by PCR, and purified again using DNA Clean Beads. The fragment-size distribution of the final libraries was examined using a Qsep400 nucleic acid fragment analyzer (BiOptic Inc., New Taipei city, Taiwan). Libraries with fragment sizes ranging from 370 to 470 bp were considered qualified. Library concentrations were measured using a Qubit fluorometer (Thermo Fisher Scientific, Waltham, MA, USA), and libraries with concentrations greater than 1 ng/μL were used for sequencing. Qualified libraries were sequenced on an Illumina NovaSeq 6000 platform (Illumina, San Diego, CA, USA). At least 6 Gb of clean sequencing data were generated for each sample.

### 2.5. Sequencing-Read Quality Control and Genome Alignment

Raw sequencing data in FASTQ format were subjected to quality control using fastp v0.23.2. Adapter-contaminated reads, reads containing excessive numbers of ambiguous nucleotides, low-quality reads with Phred quality scores below 20, and reads shorter than 50 bp after trimming were removed. The Q20 and Q30 percentages, GC content, and sequence duplication levels of the clean reads were calculated to evaluate sequencing quality. All subsequent analyses were conducted using the high-quality clean reads. The clean reads were aligned to the CAU_duck1.0 reference genome using HISAT2 v2.2.1. The corresponding reference-genome annotation file in GTF format was used to identify annotated genomic features. Alignment quality was evaluated based on the overall mapping rate, uniquely mapped read rate, and other alignment statistics. Gene-level read counts were obtained using featureCounts v2.0.1. Reads overlapping annotated exon regions were assigned to genes according to the gene_id information in the genome-annotation file. Reads that could not be assigned unambiguously to a single annotated gene were excluded from gene-level quantification.

### 2.6. Gene-Expression Quantification and Principal Component Analysis

Gene-expression levels were estimated as fragments per kilobase of transcript per million mapped fragments (FPKM) to account for differences in gene length and sequencing depth. FPKM values were used to describe and visualize the relative expression abundance of genes among samples. Raw gene-level read counts generated by featureCounts were used as input for differential-expression analysis. Differential-expression analyses and exploratory data analyses were performed in R v4.5.3. Genes with no detectable reads in all samples were removed before subsequent analyses. The raw count matrix was normalized using the median-of-ratios size-factor normalization method implemented in DESeq2 v1.50.2. For principal component analysis, normalized count data were subjected to variance-stabilizing transformation to reduce the dependence of variance on mean expression. Principal component analysis was then conducted to evaluate overall transcriptional variation, sample clustering, and potential outliers. The PCA results were visualized using ggplot2 v4.0.3, and 95% confidence ellipses were calculated for the down feather and contour feather groups.

### 2.7. Differential Expression Analysis

To compare gene-expression differences between down feather follicles and contour feather follicles while reducing bias caused by individual anatomical sites, four pairwise comparisons were established: Combination 1(C1), breast versus wing tip, Combination 2(C2), abdomen versus rump, Combination 3(C3), breast versus rump, and Combination 4(C4), abdomen versus wing tip. Differential-expression analysis for each comparison was performed using DESeq2 v1.50.2. DESeq2 models raw read counts using a negative-binomial distribution and estimates gene-specific dispersion before statistical testing. Sequencing-depth differences among samples were corrected using the size factors calculated by DESeq2. Statistical significance was evaluated using the Wald test. For all comparisons, log2 fold changes were calculated using the down feather samples relative to the contour feather samples. Therefore, a positive log2 fold-change value indicated higher expression in down feather follicles, whereas a negative value indicated higher expression in contour feather follicles. Raw *p*-values were corrected for multiple comparisons using the Benjamini–Hochberg false-discovery-rate procedure. Genes with an absolute log2 fold- change greater than 1 and an adjusted *p*-value below 0.05 were considered differentially expressed genes. Genes that met these criteria in all four pairwise comparisons were defined as core differentially expressed genes. Volcano plots were generated using ggplot2 v4.0.3. The overlap of DEGs among the four comparisons was visualized using the VennDiagram package v1.8.2. The expression patterns of the 36 core DEGs were visualized using the log2 fold-change values from the four comparisons with the pheatmap package v1.0.12.

### 2.8. KEGG Pathway Enrichment Analysis

KEGG pathway enrichment analysis was independently performed for the DEGs identified in each of the four pairwise comparisons using KOBAS 3.0. The annotated genes in the duck reference genome were used as the background gene set. DEG identifiers were converted to the corresponding KEGG identifiers before enrichment analysis. Over-representation analysis was used to determine whether the DEGs were significantly enriched in particular KEGG pathways. The resulting *p*-values were adjusted for multiple testing using the Benjamini–Hochberg method. Pathways with an adjusted *p*-value below 0.05 were considered significantly enriched. The enrichment factor was calculated as the ratio of the number of DEGs assigned to a pathway to the total number of background genes annotated to that pathway. KEGG enrichment results were visualized using ggplot2 v4.0.3 and patchwork v1.3.2. Circular plots showing the relationships between enriched pathways and their associated DEGs were generated using circlize v0.4.18. The log2 fold-change values of individual DEGs were used to indicate the direction and magnitude of differential expression.

## 3. Results

### 3.1. Overview of Transcriptome and Principal Component Analysis

**Figure 1:**
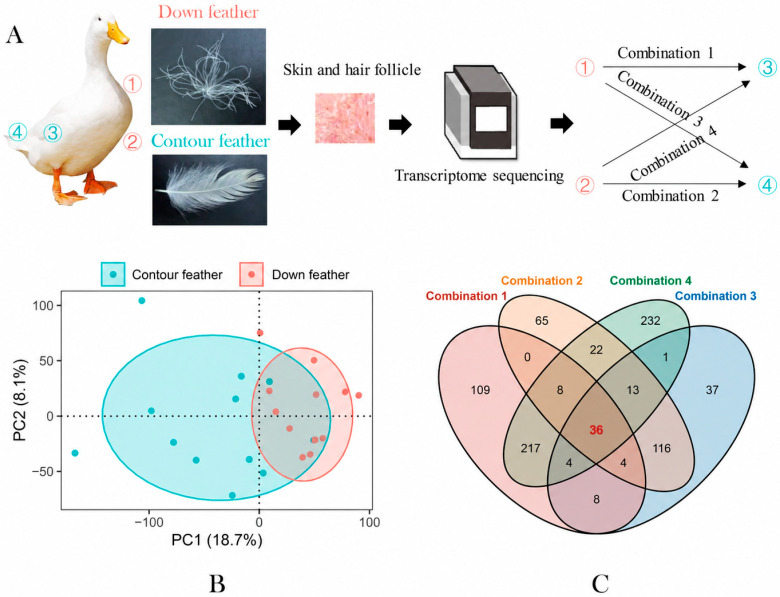
Experimental design, transcriptome quality assessment, and identification of core differentially expressed genes. (**A**) Schematic overview of the experimental design. Down feathers: breast (①) and abdomen (②), contour feathers: wing tip (③) and rump (④). (**B**) PCA of all transcriptome samples. (**C**) Venn diagram showing the overlap of DEGs identified in combinations 1–4.

Skin tissue samples containing intact follicular structures were collected from four anatomical sites of healthy adult Beijing ducks: the breast (①) and abdomen (②), which are covered by down feathers, and the wing tip (③) and rump (④), which bear contour feathers. Representative images of the two feather types are shown: down feathers are characterized by loosely branched, plumulaceous filaments lacking a coherent vane, while contour feathers possess a well-defined rachis and organized bilateral vane structure. Follicle-containing skin samples from each site were subjected to transcriptome sequencing. To systematically compare gene expression between down feather and contour feather follicles while minimizing anatomical site-specific transcriptomic variation, four pairwise comparison combinations were constructed: Combination 1 (breast vs. wing tip), Combination 2 (abdomen vs. rump), Combination 3 (breast vs. rump), and Combination 4 (abdomen vs. wing tip), encompassing all possible pairings between the two down feather sites and the two contour feather sites (Figure 1A). The first two principal components, PC1 and PC2, explained 18.7% and 8.1% of the total variation, respectively, collectively capturing approximately 26.8% of the molecular variation (Figure 1B). Down feather samples (red dots) were mainly distributed in the negative PC1 region, while contour feather samples (blue dots) clustered in the positive PC1 region. The 95% confidence ellipses of the two groups partially overlapped near the origin, reflecting their shared developmental pathways as epidermal appendages, while their respective clustering patterns revealed differences in the molecular programs supporting their functional specialization. Venn plot analysis revealed a total of 36 core DEGs across the four comparison combinations (Figure 1C). Among the group-specific DEGs, combination 4 had the most (232), followed by combination 1 (109), combination 2 (65), and combination 3 (37). In the intersection analysis of pairwise combinations, combination 1 and combination 4 have the most shared DEGs (217), while combination 1 and combination 2 have no shared DEGs that belong only to the two of them. Furthermore, the three intersections of combinations 1, 2, 3 and combinations 1, 2, 4 each contain 8 shared DEGs, and the three intersections of combinations 1, 3, 4 and combinations 2, 3, 4 each contain 4 shared DEGs.

**Figure 2:**
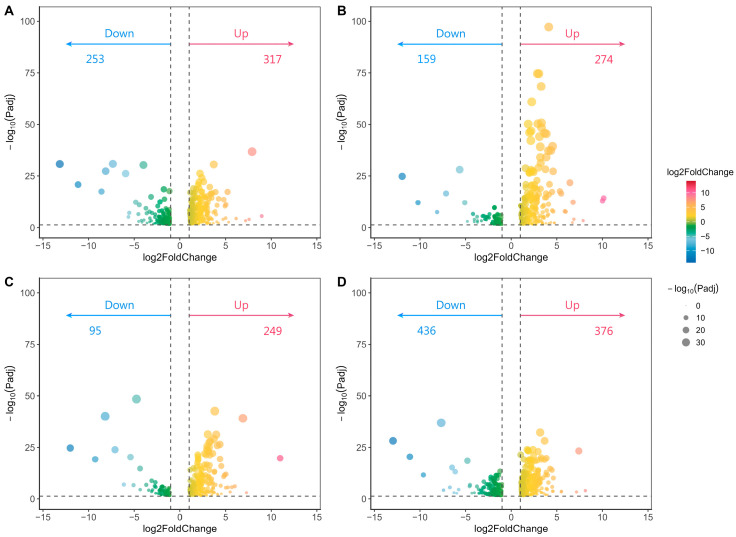
Volcano plot analysis of DEGs in combinations 1–4. (**A**): Combination 1, (**B**): Combination 2, (**C**): Combination 3 and (**D**): Combination 4. Dot size is proportional to the magnitude of −log_10_(*P*adj), and dot color ranges from blue to red, corresponding to decreasing to increasing log2FoldChange values, respectively. The horizontal dashed line denotes the significance threshold (*P*adj < 0.05), and the vertical dashed line marks the zero fold-change boundary.

In combination 1, a total of 570 DEGs were identified, including 253 downregulated genes and 317 upregulated genes (Figure 2A). Combination 2 showed 433 DEGs, including 159 downregulated genes and 274 upregulated genes (Figure 2B). Combination 3 had the fewest DEGs among all groups, 344, including 95 downregulated genes and 249 upregulated genes (Figure 2C). In contrast, combination 4 had the most DEGs, with 812 DEGs, including 436 downregulated and 376 upregulated genes (Figure 2D; Supplementary Table S1). Across all groups, most highly significant DEGs, represented by larger point sizes corresponding to higher −log10(*P*adj), were clustered near the vertical axis, while genes with extreme fold changes tended to cluster at the edges. Taken together, these results indicate significant transcriptional heterogeneity among the four groups, with C2 and C3 showing markedly skewed expression profiles and a significantly higher proportion of upregulated genes than downregulated genes. Given the established involvement of Wnt signaling in feather follicle morphogenesis, we further examined the distribution of *Wnt* family members among the DEGs in each comparison. In C1, the differentially expressed *Wnt* family genes included *WNT4*, *WNT7A*, *WNT7B*, and *WNT11*. In C4, *WNT4*, *WNT16*, *WNT11*, *WNT7B*, and *WNT10A* were identified. In contrast, only *WNT4* was detected among *Wnt* family DEGs in C2 and C3. Thus, *WNT4* was the only *Wnt* family DEG shared by all four comparisons, whereas *WNT7B* and *WNT11* were shared by the two wingtip-related comparisons, C1 and C4.

### 3.2. Expression Patterns of Core DEGs

**Figure 3:**
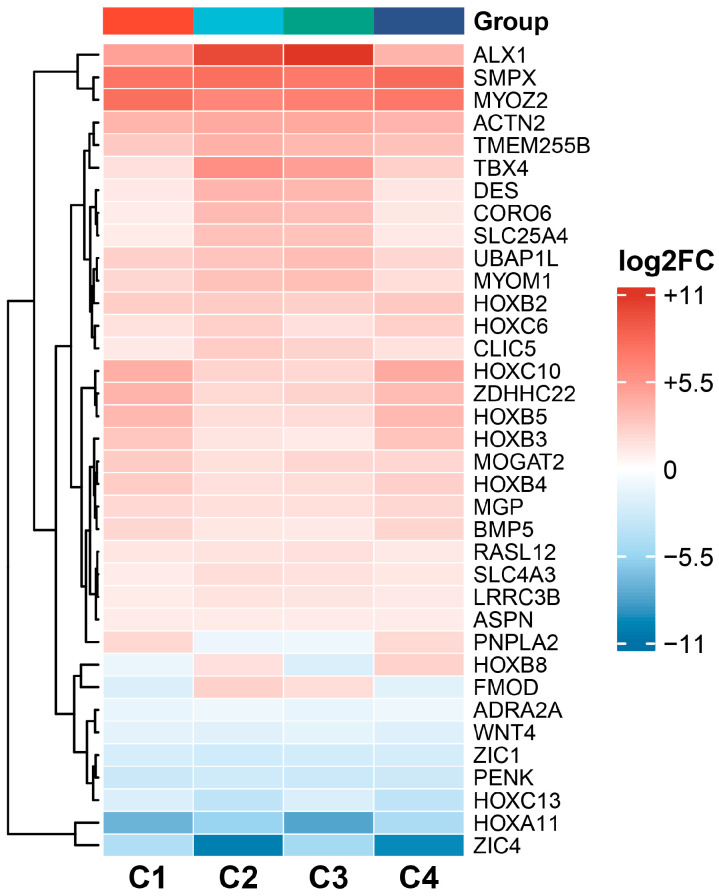
Heatmap of 36 core DEGs in combinations 1–4. Each row represents one of the 36 core DEGs and each column represents one comparison combination. Red indicates higher expression in down feather follicles and blue indicates higher expression in contour feather follicles.

The expression patterns of the 36 core differentially expressed genes were analyzed across the four paired comparison groups. The results revealed that these core genes could be divided into three major subgroups based on their differential expression profiles (Figure 3). The first category was the largest, containing most of the core differentially expressed genes from *ALX1* to *ASPN*. These genes were highly expressed in down feather follicles in all four combinations (positive log2FC), covering a diverse range of genes, including cytoskeleton and sarcomere components (*MYOZ2*, *ACTN2*, *DES*, *MYOM1*, *SMPX*), developmental transcription factors (*ALX1*, *TBX4*, *HOXB2*, *HOXC6*), other *HOXB* gene cluster members (*HOXB3*, *HOXB4*, *HOXB5*), extracellular matrix regulators (*MGP*, *ASPN*, *LRRC3B*), and lipid metabolism genes (*MOGAT2*). The upregulation levels of genes within this category varied significantly, with *ALX1*, *SMPX*, and *MYOZ2* showing the largest fold changes, with log2FC approaching +11 in some combinations. Genes such as *BMP5*, *MGP*, and *HOXB3*, however, exhibited moderate but consistent upregulation across different combinations. The second category comprises genes highly expressed in the contour feathers (negative log2FC values), meaning these genes are expressed even higher in the contour feather follicles. These include *WNT4*, *ZIC1*, *ZIC4*, *HOXC13*, *HOXA11*, *ADRA2A*, and *PENK*. Among these, *ZIC4* and *HOXA11* showed the largest negative fold changes (log2FC approaching −11 in some combinations) among all the core differentially expressed genes. The remaining genes in this class, *WNT4*, *ZIC1*, *HOXC13*, *ADRA2A*, and *PENK*, showed moderate but highly consistent downregulation across all four combinations. The third group of genes, fewer in number, consisted of *PNPLA2*, *HOXB8*, and *FMOD*. Their expression directions were inconsistent across the four combinations, suggesting relatively weaker and comparison-dependent differential expression patterns rather than stable enrichment in either feather type. These genes may be simultaneously regulated by both feather type and anatomical location, and therefore cannot be simply categorized as down feather-enriched or contour feather-enriched genes. Furthermore, compared to combinations 1 and 4 involving wingtip contour feather follicles, combinations 2 and 3 involving rump contour feather follicles showed a more systematic fold change amplitude across both up- and down-regulated gene clusters.

### 3.3. KEGG Functional Enrichment Analysis

**Figure 4:**
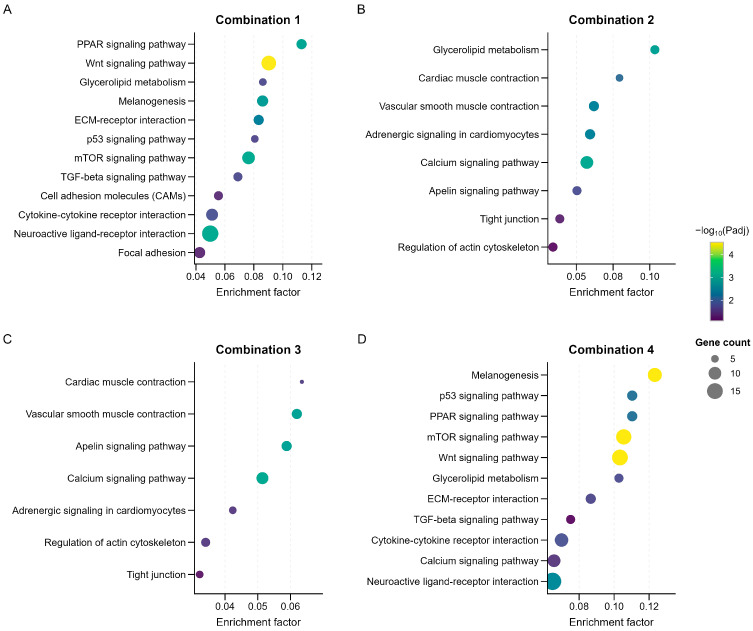
KEGG pathway enrichment analysis of DEGs for each of the four combinations. The x-axis represents the enrichment factor (the ratio of differentially expressed genes mapped to a pathway to the total number of genes in that pathway). Dot size corresponds to the number of genes involved, and dot color indicates significance as −log10(*P*adj).

KEGG pathway enrichment analysis was performed to explore the biological pathways associated with the DEGs across the four comparison groups. For Combination 1, 12 pathways were significantly enriched (Figure 4A), collectively spanning cell adhesion, metabolic regulation, and signal transduction processes. The PPAR signaling pathway exhibited the highest enrichment factor, whereas the Wnt signaling pathway showed the greatest statistical significance. Neuroactive ligand–receptor interaction contained the largest number of enriched genes. Additional pathways included glycerolipid metabolism, melanogenesis, ECM–receptor interaction, the p53, mTOR, and TGF-beta signaling pathways, cell adhesion molecules (CAMs), cytokine–cytokine receptor interaction, and focal adhesion. For Combination 2, 8 pathways were enriched (Figure 4B), predominantly associated with cardiovascular function. Glycerolipid metabolism displayed the highest enrichment factor, while the calcium signaling pathway was characterized by both a large gene count and high statistical significance. The remaining enriched pathways comprised cardiac muscle contraction, vascular smooth muscle contraction, adrenergic signaling in cardiomyocytes, the Apelin signaling pathway, tight junction, and regulation of the actin cytoskeleton. For Combination 3, 7 pathways were enriched (Figure 4C), again centered on cardiovascular processes. Cardiac muscle contraction showed the highest enrichment factor, whereas the calcium signaling pathway exhibited the largest gene count and highest significance within this group. Other enriched pathways included vascular smooth muscle contraction, adrenergic signaling in cardiomyocytes, regulation of the actin cytoskeleton, and tight junction. For Combination 4, 11 pathways were enriched (Figure 4D; Supplementary Table S2). The Wnt signaling pathway showed the highest statistical significance, while melanogenesis exhibited the highest enrichment factor. Neuroactive ligand–receptor interaction contained the largest number of enriched genes. Additional enriched pathways included the p53, mTOR, and PPAR signaling pathways, glycerolipid metabolism, ECM–receptor interaction, the TGF-beta signaling pathway, cytokine–cytokine receptor interaction, and the calcium signaling pathway. When comparing combinations 1 and 4, the enriched pathways overlapped almost completely, with two exceptions: the CAM and focal adhesion pathways were unique to combination 1, while the calcium signaling pathway was unique to combination 4. Further analysis showed that the calcium signaling pathway was significantly enriched in combinations 2, 3, and 4, but did not reach the predefined significance threshold in combination 1. Conversely, the glycerolipid metabolism was enriched in combinations 1, 2, and 4, but did not reach the predefined significance threshold in combination 3. Combinations 1 and 4 shared 10 enriched pathways, while combinations 2 and 3 shared 7. Regardless of anatomical origin, combinations consisting of down feathers from the breast or abdomen and contour feathers from the same location showed largely consistent enriched pathways. Furthermore, the pathways co-enriched by combinations 1 and 4 were fundamentally different from those co-enriched by combinations 2 and 3. Therefore, the differences in pathway enrichment between different comparison groups may reflect specific anatomical features of feather samples rather than their intrinsic biological processes.

**Figure 5:**
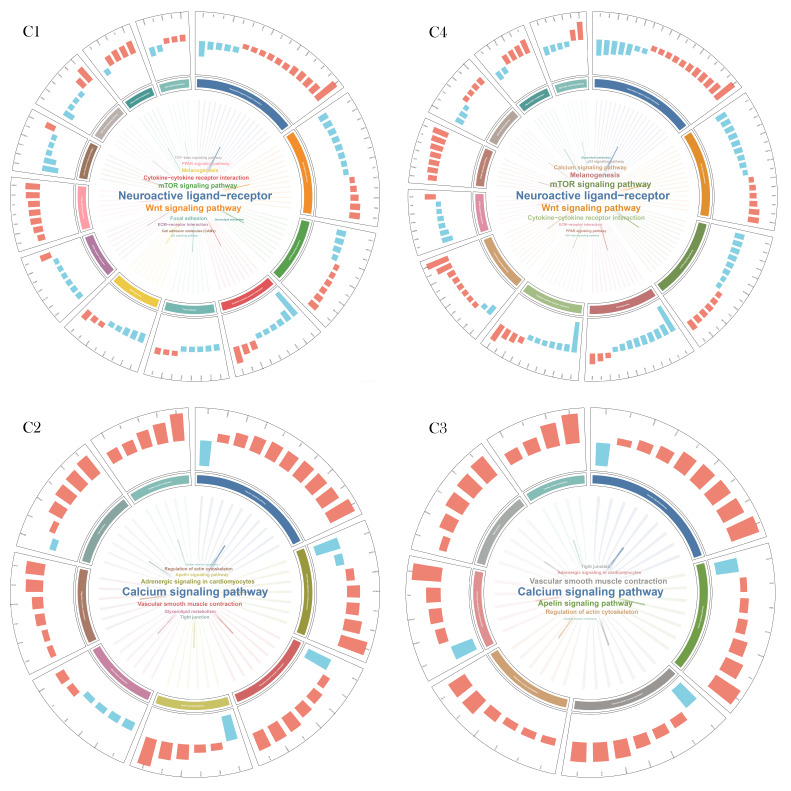
Circular representation of KEGG pathway enrichment and associated DEGs. C1: Combination 1, C2: Combination 2, C3: Combination 3 and C4: Combination 4. The central word cloud highlights enriched pathways identified from the analysis, with font size reflecting the degree of enrichment. Surrounding bar plots show the log2 fold change of individual DEGs mapped to each pathway. Red indicates higher expression in down feather follicles and blue indicates higher expression in contour feather follicles.

At the gene level, the composition of enriched pathways was visualized using a circular plot (Figure 5). In combination 1, the Wnt signaling pathway and neuroactive ligand–receptor interaction pathway contained the largest numbers of annotated genes, most of which exhibited mixed patterns of up- and down-regulation. Similar patterns were observed in combination 4. Notably, all genes enriched in the PPAR signaling pathway were upregulated in both combinations 1 and 4. In contrast, combinations 2 and 3 displayed distinct pathway profiles, with the calcium signaling pathway containing the greatest number of annotated genes. Moreover, most DEGs in these two comparisons were upregulated, including all genes enriched in the cardiac muscle contraction and regulation of actin cytoskeleton pathways. Further examination identified eight DEGs that were significantly enriched in at least one pathway. *WNT4* was consistently enriched in the Wnt signaling pathway, mTOR signaling pathway, and melanogenesis pathway in both combinations 1 and 4. *BMP5* and *FMOD* were co-enriched in the TGF-β signaling pathway in combinations 1 and 4, consistent with their established roles in extracellular matrix remodeling. Similarly, *MOGAT2* and *PNPLA2* were co-enriched in the glycerolipid metabolism pathway in combinations 1, 2, and 4. *ADRA2A* and *PENK* were co-enriched in the neuroactive ligand–receptor interaction pathway in combinations 1 and 4. Finally, *SLC25A4* was among the DEGs mapped to the calcium signaling pathway in combinations 2, 3, and 4, but was not identified among the pathway-associated DEGs in combination 1. This pattern aligns with the enrichment analysis and provides a potential explanation, at the single-gene level, for the calcium signaling pathway enrichment detected in groups 2, 3, and 4.

## 4. Discussion

In this study, multi-site transcriptomic comparisons identified 36 DEGs shared across four contrasts between the sampled contour and down feather follicles. Rather than indicating a single master regulator, the results point to two partially distinct transcriptional states. Based on their consistent differential expression patterns and potential biological relevance, we first focused on eight representative candidate genes. Down feather follicles were characterized by higher expression of *HOXC6*, *HOXC10*, *TBX4*, and *BMP5*, whereas contour feather follicles showed higher *WNT4*, *ZIC1*, *ZIC4*, and *HOXC13* expression. Together with pathway enrichment, these patterns suggest that differences between the sampled follicle types may arise from the combined effects of regional identity, epithelial–mesenchymal signaling, early patterning, keratinization, and the follicular microenvironment.

### 4.1. Down Feather Follicles Show a Positional and Mesenchymal-Associated Expression Pattern

The higher expression of *HOXC6*, *HOXC10*, and *TBX4* in down feather follicles is consistent with, and extends, previous findings. A transcriptomic comparison of duck plumulaceous and flight feathers also identified *HOXC10* and *TBX4* among genes associated with plumulaceous feathers across developmental stages [24]. This agreement across species and feather types suggests that their expression is not merely an isolated observation in the present dataset. However, *HOX* genes are also linked to regional skin identity: *HOX* expression varies among skin regions [33,34,35], *HOXC4-10* expression differs between regenerating dorsal follicles and resting ear follicles in mice [36], and ectopic activation of *HOXC* genes is associated with crest formation in Polish chickens [37,38]. Therefore, our results are best interpreted as evidence that down feather follicles retain a distinct positional or mesenchymal transcriptional state, rather than as proof that *HOXC6* or *HOXC10* directly determines down feather morphology.

*TBX4* strengthens this interpretation because it is a mesenchymal developmental regulator [39,40,41,42,43,44]. Given that dermal papilla signals influence barb and barbule organization through pathways including Wnt, BMP, and Shh [9,45], elevated *TBX4* may mark a mesenchymal environment that favors the loose, radially organized structure of down feathers. In parallel, *BMP5* was higher in down feather follicles, and additional BMP/TGF-beta-related genes (*BMP7*, *TGFB3*, *INHBB*, and *ACVR1C*) contributed to the enriched TGF-beta-related signal. Previous studies in chickens have established roles for BMP-related signaling in feather placode formation and barb and rachis patterning [20,46,47,48,49,50,51], although *BMP5* itself has not been directly tested in feather-type differentiation. Evidence from geese further supports a broader, stage-dependent involvement of *BMP* family genes in feather follicle development. In embryonic dorsal skin of *Anser anser*, *BMP7* expression decreased from the primary feather follicle stage toward the later stages of secondary follicle development, whereas *BMP6* was downregulated between the early and later stages of secondary follicle development in *Anser cygnoides* [52,53]. A comparative transcriptomic analysis of *Anser anser* and *Anser cygnoides* also detected higher *BMP7* expression in embryonic feather-follicle skin of *Anser anser* [54]. These goose studies do not establish a *BMP5*-specific function or a direct relationship between *BMP* family expression and down-feather identity, but they indicate that *BMP* family genes exhibit developmental-stage- and species-associated expression differences in waterfowl feather follicles.

We therefore propose that the down-feather-associated pattern reflects a regional mesenchymal state accompanied by altered BMP/TGF-beta-related signaling. This hypothesis should be tested by localizing *TBX4* and *BMP5* within the dermal papilla and surrounding mesenchyme and by perturbing BMP signaling.

### 4.2. Contour Feather Follicles Show Stronger Patterning and Keratinization-Associated Signals

In contrast, *WNT4* expression in contour feather follicles was approximately four times that in down feather follicles. The Wnt signaling pathway also reached the enrichment threshold in C1 and C4, both involving wingtip contour feather follicles. This result is compatible with the greater structural coordination required for an ordered rachis-vane-barb hierarchy. Previous experimental studies in chickens showed that the spatial distribution and intensity of Wnt signaling influence directional barb growth and that inhibition of *WNT3A* can shift bilaterally symmetric vane structures toward radially symmetric downy feathers [55,56,57]. Comparative transcriptomic studies have also reported Wnt-pathway differences among feather types [28,58], and *WNT4*, *WNT5a*, and *WNT6* show dynamic epithelial expression during feather regeneration [59]. Our data therefore identify *WNT4* as a plausible marker or contributor to contour feather morphogenesis. Nevertheless, transcript abundance and KEGG enrichment do not demonstrate increased pathway activity, and the specific role of *WNT4* remains to be validated. The *Wnt* family DEG pattern observed here can be understood within the known spatiotemporal diversity of Wnt ligands during feather morphogenesis. In the present dataset, *WNT4* represented the most consistent *Wnt* family DEG, whereas other Wnt ligands appeared in a comparison-dependent manner, suggesting that Wnt-related differences between down- and contour-feather-associated regions may involve regionally modulated signaling rather than a uniform change in a single ligand. Previous work in chicken feather development showed that *WNT7A* expression becomes progressively restricted from the feather tract epithelium to the primordia domain, the primordia border, and finally the posterior feather bud, and perturbation of *WNT7A* disrupts anterior–posterior organization and proximal–distal elongation [10]. Other Wnt members also display distinct compartment-associated patterns: *WNT1* and *WNT3A* become restricted to the placodal epithelium and later the elongated distal bud epidermis, whereas *WNT5A* and *WNT11* are enriched in inter-tract or interprimordia regions and later appear in the elongated distal bud dermis [60]. During feather regeneration, Wnt signaling is also highly active, and Wnt ligands including *WNT4*, *WNT5A*, and *WNT6* show dynamic expression changes, further supporting a role for Wnt-ligand diversity in follicle regeneration [59]. These studies indicate that individual Wnt ligands are associated with different epithelial, dermal, and developmental contexts during feather formation. Therefore, the *Wnt* family differences observed in our comparisons may reflect region-associated differences in follicular patterning and epithelial–mesenchymal signaling. Because all samples in this study were collected at the same age, these data should be interpreted as spatial, region-associated transcriptomic differences rather than direct evidence of temporal Wnt dynamics.

The higher expression of *ZIC1*, *ZIC4*, and *HOXC13* in contour feather follicles provides a complementary signal. *ZIC1* and *ZIC4* are expressed in dermal feather-forming regions and decrease after suppression of SHH signaling [61]. In addition, *ZIC1* is enriched in large feather primordia and can initiate an invagination step during scale-to-feather conversion, although it is insufficient to produce mature follicles [62]. These findings fit our observation that contour feathers, particularly wingtip feathers, require more elaborate patterning than down feathers. *HOXC13* may act at a later differentiation stage: in mammals, *HOXC13* disruption causes hair defects and *HOXC13* regulates keratin genes [63,64,65]. The cross-species evidence does not establish an avian mechanism, but it supports the hypothesis that increased *HOXC13* expression is related to the greater structural stability and keratinization of contour feathers.

### 4.3. A Signaling-Balance Model May Explain the Divergence Between Feather Types

Taken together, the results suggest a working model in which contour and down feather follicles differ in the balance and spatial organization of conserved morphogenetic programs rather than in the presence or absence of a single pathway. In the sampled contour follicles, higher *WNT4* together with *ZIC1*, *ZIC4*, and *HOXC13* may support coordinated patterning and terminal structural differentiation. In the sampled down follicles, higher *HOXC6*, *HOXC10*, *TBX4*, *BMP5*, and other BMP/TGF-beta-related genes may reflect a positional and mesenchymal state favoring radial barb organization. This model is consistent with the known dependence of feather morphology on epithelial–mesenchymal interactions [9,45,46,47,48,49,50,51,66], but it remains a hypothesis: the current data do not show that *WNT4* and *BMP5* act antagonistically, nor do they establish whether the observed differences originate in the epithelium, dermal papilla, or other cell populations.

Some pathway annotations should also be interpreted as secondary clues rather than mechanistic conclusions. For example, *WNT4* was mapped to mTOR signaling and melanogenesis in KEGG. The mTOR mapping may reflect differences in proliferation, metabolism, or growth status, given interactions between mTOR, BMP, and Wnt signaling in mammalian hair follicles [67]. The melanogenesis mapping may reflect shared Wnt-related nodes rather than pigmentation differences. These annotations can guide follow-up analyses but should not be used to infer pathway activation from transcriptomic enrichment alone.

On the basis of the present transcriptomic evidence, these genes should be distinguished as feather-type-associated markers rather than established causal regulators. We prioritize *WNT4* and *BMP5* as candidate regulators for subsequent causal-validation experiments because they represent the Wnt and BMP/TGF-β signaling axes, respectively. *ZIC1*, *ZIC4*, and *HOXC13* are considered secondary regulatory candidates, whereas *HOXC6*, *HOXC10*, and *TBX4* require particularly cautious interpretation because of their possible association with anatomical positional identity. Demonstrating causality will require spatial localization and gain- or loss-of-function experiments showing that manipulation of these genes alters rachis formation, barb organization, or radial versus bilateral feather symmetry.

### 4.4. Other Core DEGs May Reflect Differences in the Follicular Microenvironment

The remaining core DEGs may help explain how the follicular microenvironment supports the two morphologies. ECM-related genes *ASPN*, *FMOD*, and *MGP* were accompanied by enrichment of ECM–receptor interaction, focal adhesion, and cell-adhesion-related pathways. Because contour feathers form an ordered rachis-vane-barb hierarchy whereas down feathers have a looser radial arrangement, differences in matrix remodeling, adhesion, and mechanical support are biologically plausible [68,69,70]. These changes may be part of the morphogenetic process, but they may also reflect differences in the proportions or states of mesenchymal cell populations.

Similarly, *ACTN2*, *DES*, *MYOM1*, *MYOZ2*, *SMPX*, and *CORO6* were associated with calcium signaling, actin-cytoskeleton regulation, and vascular smooth muscle contraction pathways. Rather than indicating the formation of typical smooth muscle tissue, these signals may originate from follicle-associated muscle, vascular or interstitial cells, or cytoskeletal remodeling within follicular cells [20,71,72].

The calcium signaling pathway reached the significance threshold in combinations 2, 3, and 4, but not in combination 1. This non-significant result should not be interpreted as the biological absence or complete inactivity of calcium signaling in the breast or wingtip follicles. Because *KOBAS* gene-list enrichment analysis evaluates whether pathway-associated genes are statistically over-represented among the input DEGs relative to the background gene set, failure to pass the corrected significance threshold indicates insufficient statistical over-representation rather than the absence of the underlying biological process [73]. In developing chicken skin, calcium-channel components, including *CACNA1D*, exhibit spatiotemporally restricted epithelial expression during regional skin differentiation [74]. Synchronized Ca^2+^ oscillations have also been observed during chicken feather elongation, where they expand progressively and coordinate SHH- and Wnt-dependent mesenchymal cell migration [75]. These findings support the view that calcium-related transcriptional signals vary with anatomical context, developmental state, and cellular activity. In the present experimental design, the breast was also included in combination 3 and the wingtip in combination 4, both of which showed significant enrichment of the calcium signaling pathway. Therefore, the non-significant result in combination 1 cannot be attributed to either anatomical site alone and is more consistent with the breast-versus-wingtip comparison producing insufficient over-representation of calcium-signaling-associated DEGs to pass the predefined statistical threshold.

A similar interpretation applies to glycerolipid metabolism. The glycerolipid metabolism pathway reached the significance threshold in combinations 1, 2, and 4, but not in combination 3. Previous studies support the involvement of glycerolipid-related processes in waterfowl feather follicle development, while also indicating that their detection varies among developmental comparisons. A study examining the Pekin duck microRNAome across six developmental stages identified glycerolipid metabolism among the lipid synthesis- and metabolism-related pathways associated with feather follicle development and proposed a possible relationship with lipid formation on the feather surface [76]. In embryonic goose skin, glycerolipid metabolism was reported among the pathways enriched in the E10-versus-E28 comparison, indicating that the prominence of this pathway can vary with developmental context [77]. In the present dataset, *MOGAT2* and *PNPLA2* were mapped to the glycerolipid metabolism pathway in combinations 1, 2, and 4, but not in combination 3. Moreover, combination 3 contained the fewest DEGs and showed the weakest overall pathway enrichment among the four comparisons. Because the breast and rump samples each participated in another comparison in which glycerolipid metabolism was significantly enriched, its non-significant enrichment in combination 3 is unlikely to indicate the absence of glycerolipid metabolism from either anatomical region. Instead, the breast-versus-rump comparison appears to have provided insufficient over-representation of glycerolipid-metabolism-associated DEGs after multiple-testing correction. Thus, this result is more appropriately interpreted as a comparison-specific difference in the strength of the glycerolipid-related transcriptional signal.

The enrichment of lipid-related signals was further supported by the PPAR signaling pathway. In combinations 1 and 4, this pathway was significantly enriched, and all DEGs mapped to it showed positive log2 fold changes, indicating higher expression in the breast and abdominal down-feather samples, respectively, than in the wingtip contour-feather samples. Experimental studies in mammalian skin have shown that PPAR*β*/*δ* activation promotes keratinocyte differentiation, triglyceride accumulation, and permeability-barrier homeostasis, whereas conditional deletion of PPAR*γ* in mouse hair-follicle stem cells disrupts the maintenance of the follicular unit [78,79]. In avian studies, PPAR signaling has been identified among the commonly enriched pathways in embryonic goose skin feather follicles [54], while lipid synthesis- and metabolism-related pathways have also been implicated in Pekin duck feather-follicle development and were proposed to contribute to lipid formation on the feather surface [76]. Together, these observations suggest that the coordinated higher expression of PPAR-signaling-associated DEGs in combinations 1 and 4 may reflect differences in lipid handling, epidermal differentiation, or local skin homeostasis between down-feather-associated skin and wingtip contour-feather-associated skin. However, because both comparisons shared the wingtip as the contour-feather comparator, this pattern cannot yet be attributed exclusively to down-feather identity and may also contain an anatomical-site-specific component. Moreover, the consistent positive fold changes of pathway-mapped DEGs support a down-feather-associated transcriptional pattern but do not by themselves demonstrate activation of the PPAR pathway or a direct role in determining down-feather morphology. Lipid-metabolism-related genes *PNPLA2* and *MOGAT2* and neuroactive genes *PENK* and *ADRA2A* may indicate differences in physiological state, surface properties, or local neural and neuroendocrine regulation [76,77,80,81]. At present, these findings are most useful as hypotheses for cell-type-resolved analysis rather than as evidence of direct control over feather-type differentiation.

Comparable pathway-level patterns have also been reported during goose feather follicle development. In *Anser anser*, ECM–receptor interaction was one of the most strongly enriched pathways during both the transition from primary to secondary feather follicle formation and the subsequent development of secondary follicles [52]. In *Anser cygnoides*, stage-associated DEGs were assigned to ECM–receptor interaction, focal adhesion, regulation of the actin cytoskeleton, calcium signaling, Wnt signaling, and neuroactive ligand–receptor interaction pathways [53]. Direct comparison of embryonic feather-follicle skin between *Anser anser* and *Anser cygnoides* further identified calcium signaling, PPAR signaling, glycerolipid metabolism, and ECM–receptor interaction among the major enriched pathways, while the relative enrichment of several pathways differed between the two species [54]. More recently, a five-stage comparison between Zhedong white and Hungarian white geese identified common DEGs associated with neuroactive ligand–receptor interaction and calcium signaling, together with co-expression modules enriched in ECM–receptor interaction, PPAR, Wnt, mTOR, and calcium signaling pathways [82]. These overlaps support considering the ECM, cytoskeletal, lipid-metabolic, calcium-associated, and neuroactive signatures identified in our study as candidate components of the avian feather-follicle microenvironment rather than as annotations restricted to ducks. Nevertheless, because the goose studies compared embryonic developmental stages or breeds rather than adult contour and down feather follicles, they do not demonstrate that these pathways directly specify feather type.

### 4.5. Limitations and Future Directions

Several limitations should be considered. First, retaining DEGs shared across all four comparisons reduced the influence of anatomical-site-specific variation, but contour and down feather follicles could not be collected from identical sites. Regional effects therefore cannot be fully excluded, particularly for *HOX*-related genes. Second, bulk transcriptomics cannot distinguish cell-intrinsic regulatory changes from shifts in cellular composition. Third, residual differences in follicle growth or cycling stage may contribute to the expression patterns. Fourth, pathway enrichment identifies over-represented gene sets but does not directly demonstrate pathway activation, inhibition, or causal relationships. Finally, several interpretations, especially for *HOXC13* and mTOR-related signals, rely partly on cross-species evidence.

Future work should test the proposed model in a stepwise manner. In situ hybridization, immunostaining, or spatial transcriptomics should determine whether *HOXC6*, *HOXC10*, *TBX4*, *BMP5*, *WNT4*, *ZIC1*, *ZIC4*, and *HOXC13* are localized to the epithelium, dermal papilla, or surrounding mesenchyme. Single-cell transcriptomics could separate changes in cell composition from changes within specific populations. Protein-level assays and pathway-activity measurements are needed to evaluate Wnt and BMP/TGF-beta signaling states. Finally, gene editing, organ culture, or signaling perturbation experiments should test whether manipulating *WNT4* or *BMP5* changes barb organization and whether states associated with *HOXC6*, *HOXC10*, *TBX4*, *ZIC1*, *ZIC4*, and *HOXC13* influence radial versus ordered feather structures. These experiments would distinguish feather-type-associated markers from causal regulators and clarify how regional identity and morphogenetic signaling interact during feather differentiation.

## 5. Conclusions

In summary, this study employed multi-regional comparative transcriptomic analyses to characterize gene expression differences and associated biological pathways between down feather- and contour feather-associated skin regions in ducks. A set of genes consistently differentially expressed across multiple comparisons was identified, along with pathways potentially involved in feather-type differentiation. These findings provide a transcriptomic resource for further investigation of molecular differences between feather types and offer candidate genes for future functional studies of feather development.

## Data Availability

The data presented in this study are available on request from the corresponding authors.

## Supplementary Materials

The following supporting information can be downloaded at: https://www.mdpi.com/article/10.3390/ani16142267/s1. Table S1. The list of differentially expressed genes. Table S2. Enrichment analysis results.

## Abbreviations

The following abbreviations are used in this manuscript:

DEGs	Differentially expressed genes
CAMs	Cell adhesion molecules
PCA	Principal component analysis
C1	Combination 1
C2	Combination 2
C3	Combination 3
C4	Combination 4
